# Supplementary material for: Identification of core genes involved in the response of Apocynum venetum to salt stress based on transcriptome sequencing and WGCNA
Source: PLoS One. 2024 Apr 30;19(4):e0300277. doi: 10.1371/journal.pone.0300277 (PMC11060595; doi:10.1371/journal.pone.0300277)
Supplement: Attachment — Submitted filename: Abstract.pdf [file pone.0300277.s001.pdf]

Comments to the authors

Abstract

1. Methods of salt stress are briefly emphasized in the abstract

Some conjunctions can be omitted if the number of words is limited, like "Herein , Finally". Please check the presentation carefully.

This sentence: "TRINITY_DN102_c0_g1, TRINITY_DN3073_c0_g1 and TRINITY_DN6732_c0_g1 related to serine carboxypeptidase, SOS signaling pathway and heat shock transcription factor in the red module were determined to be the core genes in the red module" , the phrase of "in the red module" appear two times.

Introduction

1. Please check the statement of the article, some sentences are repeated. For example"In summary, scholars in China and around the world have carried out many studies on the medicinal components, genetic diversity, and salt tolerance of *A. venetum*, but the molecular mechanism of the salt tolerance of *A. venetum* is still unclear, and more in-depth research is needed. "

Because of the previous similar description, here can be rephrased.

Like: Although there are some research results inthe medicinal components, genetic diversity, and salt tolerance of *A. venetum*, it is very necessary to conduct in-depth research in the molecular mechanism of the salt tolerance of *A. venetum*.

Results

1. There are some mistakes of words. Like: "All indexes of the original transcriptome sequencing data met the data requirements and couble be spliced and assembled in the next step".

2. In Table 1, there are two columns of "raw reads"

3. In the part of Correlation analysis, does the value of the correlation coefficient represent the strength of the correlation? Please state them in the notes of Fig or in the corresponding paragraph.

4. Please state them in the notes of Figures, like Fig8, 9, 11,12, What do dots, lines or columns represent in the picture?

5. In the results section of the qRT-PCR analysis, Figure 12 displayed qRT-PCR results for 15 genes, but only results for 9 genes are stated in the text. This

discrepancy should be rectified to ensure consistency between the number of genes presented in the figure and those discussed in the text.

Discussion

1. In the concluding section, the author mentions "TRINITY_DN9926_c0_g1 and TRINITY_DN7962_c0_g1, which may be new genes in *A. pseudotensis*, and their roles in response to salt stress need to be further explored." It appears that these new genes were discussed in the context of *A. pseudotensis*, while the experimental material used was *Apocynum venetum*.

2. "The higher the connectivity of one gene in the WGCNA is, the higher the degree of connection between this gene and other genes and the higher the importance of this gene; such a gene is usually a core gene. " whose opinion is it? Please add the references or explain it.

Materials

1. Is this a repetition?

Wild *A. venetum* plants with good growth were divided into two groups, the salt stress treatment group and the control group, and each treatment was repeated 3 times. According to the pre-experiment, 300 mmol/L was selected as the stress concentration for the transcriptome test.

Wild Apocaf plants with good growth were divided into two groups, the salt stress treatment group and the control group, and each treatment was repeated 3 times.

2. Please add the collection site or planting site. If it is wild, how to select the salt stress test site and control test site? If it is grown, what salt you added and how much, please describe clearly and briefly in the part of Abstract.

3. How many test treatments? How many repetitions per process? What is the sample size used in the test?

4. In the transcriptomics section, it is essential to specify whether the sequencing work was conducted by a sequencing company. If so, this information should be explicitly stated in the manuscript. Providing this detail in the article is crucial for transparency and reproducibility, as it allows other researchers to understand the data generation process and the involvement of an external sequencing service provider.

5. The software or analysis platform, like "DESeq software" requires a version number or a reference.

6. Why did you choose these methods in your research, like WGCNA technique. So please state them in the part of Methods.

7. This part of "**Screening of key salt-tolerance genes of *Apocynum venetum* by the WGCNA technique…** ", is it misplaced? The result is not described here

8. It is shown in the result that Go analysis was done twice, why did you do so, please state in the method.

9. How to do the "**Co-expression network analysis"?** please state in the method.
