# Supplementary material for: Identification of core genes involved in the response of Apocynum venetum to salt stress based on transcriptome sequencing and WGCNA
Source: PLoS One. 2024 Apr 30;19(4):e0300277. doi: 10.1371/journal.pone.0300277 (PMC11060595; doi:10.1371/journal.pone.0300277)
Supplement: Attachment — Submitted filename: Review_Shaba.docx [file pone.0300277.s002.docx]

**Journal of Plant Diseases and Protection**

**Title**: Identification of core genes involved in the response of Apocynum venetum to salt stress based on transcriptome sequencing and WGCNA

**Id-** PONE-D-23-25332

**General Comments:**

Presented study nicely planned and implemented will be useful for further related scholars.

The article accepted with some minor revision.

Please find below the specific points we would like you to addres

**Comments**

1. In the Material and Methods section, kindly ensure to explicitly mention the tool or software utilized for the calculation of FPKM in the determination of gene expression values
2. The Material and Methods section appears to be incomplete. We kindly request you to incorporate the following:

- Clearly state the developmental stage of the plant at which the sample was collected for transcriptome analysis
- Provide a detailed introduction to Co-expression network analysis, along with pertinent information regarding related packages such as WGCNA, in the Material and Methods section.
- Elaborate on the techniques and tools employed for the formation of Gene Ontology annotations

3. For enhanced clarity and comprehension, we recommend including a supplementary table presenting the correlation coefficients of gene pairs derived from the WGCNA analysis.
4. Please provide a comprehensive explanation and specification of the twelve modules obtained from the WGCNA analysis.

I believe that these revisions will significantly contribute to the comprehensiveness and clarity of your research

Thank you
