# Supplementary material for: Identification of core genes involved in the response of Apocynum venetum to salt stress based on transcriptome sequencing and WGCNA
Source: PLoS One. 2024 Apr 30;19(4):e0300277. doi: 10.1371/journal.pone.0300277 (PMC11060595; doi:10.1371/journal.pone.0300277)
Supplement: Attachment — Submitted filename: Reply to the editors comments.docx [file pone.0300277.s003.docx]

**Reply to the editor's comments**

When submitting your revision, we need you to address these additional requirements.

1. Please ensure that your manuscript meets PLOS ONE's style requirements, including those for file naming. The PLOS ONE style templates can be found at

https://journals.plos.org/plosone/s/file?id=wjVg/PLOSOne_formatting_sample_main_body.pdf and

https://journals.plos.org/plosone/s/file?id=ba62/PLOSOne_formatting_sample_title_authors_affiliations.pdf

Reply: We have revised the manuscript format as requested.

2. PLOS requires an ORCID iD for the corresponding author in Editorial Manager on papers submitted after December 6th, 2016. Please ensure that you have an ORCID iD and that it is validated in Editorial Manager. To do this, go to ‘Update my Information’ (in the upper left-hand corner of the main menu), and click on the Fetch/Validate link next to the ORCID field. This will take you to the ORCID site and allow you to create a new iD or authenticate a pre-existing iD in Editorial Manager. Please see the following video for instructions on linking an ORCID iD to your Editorial Manager account: https://www.youtube.com/watch?v=_xcclfuvtxQ

Reply: The ORCID of the first author of the manuscript is 0009-0007-2631-7566. And we have linked ORCID to our editorial manager account.

3. Your ethics statement should only appear in the Methods section of your manuscript. If your ethics statement is written in any section besides the Methods, please move it to the Methods section and delete it from any other section. Please ensure that your ethics statement is included in your manuscript, as the ethics statement entered into the online submission form will not be published alongside your manuscript.

Reply: We have written ethical statements in our materials and methods.

4. Please review your reference list to ensure that it is complete and correct. If you have cited papers that have been retracted, please include the rationale for doing so in the manuscript text, or remove these references and replace them with relevant current references. Any changes to the reference list should be mentioned in the rebuttal letter that accompanies your revised manuscript. If you need to cite a retracted article, indicate the article’s retracted status in the References list and also include a citation and full reference for the retraction notice.

Reply: There are no references to the retracted papers in the manuscript.

Two articles have been added to the references and have been marked in the list.

**Respond to reviewer comments**

**Reviewer #1:**
1.Is the manuscript technically sound, and do the data support the conclusions?
Yes.
2.Has the statistical analysis been performed appropriately and rigorously?
Yes. In correlation analysis, it is better to provide a significant difference analysis in addition to the correlation coefficient
3.Have the authors made all data underlying the findings in their manuscript fully available?
Yes.
4.Is the manuscript presented in an intelligible fashion and written in standard English?
The English writing is good, but the article needs further careful proofreading to avoid typos.
Review:
Abstract
1. Methods of salt stress are briefly emphasized in the abstract
Some conjunctions can be omitted if the number of words is limited, like “Herein ，Finally”. Please check the presentation carefully.
This sentence: “TRINITY_DN102_c0_g1, TRINITY_DN3073_c0_g1 and TRINITY_DN6732_c0_g1 related to serine carboxypeptidase, SOS signaling pathway and heat shock transcription factor in the red module were determined to be the core genes in the red module” , the phrase of “in the red module” appear two times.

Reply: It has been revised in the manuscript.

Introduction
1.Please check the statement of the article, some sentences are repeated. For example“In summary, scholars in China and around the world have carried out many studies on the medicinal components, genetic diversity, and salt tolerance of A. venetum, but the molecular mechanism of the salt tolerance of A. venetum is still unclear, and more in-depth research is needed. ”
Because of the previous similar description, here can be rephrased.
Like: Although there are some research results inthe medicinal components, genetic diversity, and salt tolerance of A. venetum, it is very necessary to conduct in-depth research in the molecular mechanism of the salt tolerance of A. venetum.

Reply: These questions have been revised in the manuscript.

Results
1.There are some mistakes of words. Like: “All indexes of the original transcriptome sequencing data met the data requirements and couble be spliced and assembled in the next step”.

Reply: Redundant data has been deleted from the received manuscript.

2.In Table 1, there are two columns of “raw reads”

Reply: Duplicate data has been removed from the manuscript.

3.In the part of Correlation analysis, does the value of the correlation coefficient represent the strength of the correlation? Please state them in the notes of Fig or in the corresponding paragraph.

Reply:This issue has been addressed in the manuscript.

4.Please state them in the notes of Figures, like Fig8, 9, 11,12, What do dots, lines or columns represent in the picture?

Reply: The points, lines, and columns in Figures 8, 9, 11, and 12 have been explained in the comments in the figure.

5.In the results section of the qRT-PCR analysis, Figure 12 displayed qRT-PCR results for 15 genes, but only results for 9 genes are stated in the text. This discrepancy should be rectified to ensure consistency between the number of genes presented in the figure and those discussed in the text.

Reply: The number of genes has been modified in the manuscript

Discussion
1.In the concluding section, the author mentions "TRINITY_DN9926_c0_g1 and TRINITY_DN7962_c0_g1, which may be new genes in A. pseudotensis, and their roles in response to salt stress need to be further explored." It appears that these new genes were discussed in the context of A. pseudotensis, while the experimental material used was Apocynum venetum.

Reply: TRINITY_DN9926_c0_g1 and TRINITY_DN7962_c0_g1 are genes identified in Apocynum and have been modified in the manuscript.

2.“The higher the connectivity of one gene in the WGCNA is, the higher the degree of connection between this gene and other genes and the higher the importance of this gene; such a gene is usually a core gene. ” whose opinion is it? Please add the references or explain it.

Reply: This issue has been revised and explained in the manuscript.

Materials and methods
1.Is this a repetition?
Wild A. venetum plants with good growth were divided into two groups, the salt stress
treatment group and the control group, and each treatment was repeated 3 times. According to the pre-experiment, 300 mmol/L was selected as the stress concentration for the transcriptome test.
Wild Apocaf plants with good growth were divided into two groups, the salt stress treatment group and the control group, and each treatment was repeated 3 times.

Reply: The design of repetition in the teast has been revised in the manuscript.

2.Please add the collection site or planting site. If it is wild, how to select the salt stress test site and control test site? If it is grown, what salt you added and how much, please describe clearly and briefly in the part of Abstract.

Reply: We have added the collection site or planting site information to the Materials and Methods section.

3.How many test treatments? How many repetitions per process? What is the sample size used in the test?

Reply: After 3 days of acclimatization in the laboratory, plants with good growth were selected to the salt stress treatment.To ensure the ability to conduct statistical analyses, three biological repli-cates were used for the transcriptome sequencing analysis and three biological replicates were used for qRT-PCR analysis.

4.In the transcriptomics section, it is essential to specify whether the sequencing work was conducted by a sequencing company. If so, this information should be explicitly stated in the manuscript. Providing this detail in the article is crucial for transparency and reproducibility, as it allows other researchers to understand the data generation process and the involvement of an external sequencing service provider.

Reply: The transcriptome sequencing company is listed in the acknowledgments of the manuscript. Transcriptome sequencing company for OE biotech Co., Ltd. (Shanghai, China). Transcriptome data were analyzed for the manuscript authors.

5.The software or analysis platform， like "DESeq software" requires a version number or a reference.

Reply: The version number of the software was added to the manuscript as required by the reviewer.

6.Why did you choose these methods in your research, like WGCNA technique. So please state them in the part of Methods.

Reply: The reasons and significance of choosing WGCNA technology are added to the Methods section.

7.This part of “Screening of key salt-tolerance genes of Apocynum venetum by the WGCNA technique… ”, is it misplaced? The result is not described here

Reply: “Screening of key salt-tolerance genes of Apocynum venetum by the WGCNA technique” is the title of this section .Data preprocessing and construction of the sample level clustering tree is part of the WGCNA analysis.

8.It is shown in the result that Go analysis was done twice, why did you do so, please state in the method.

Reply: A total of two GO analyses were used, one for genes at all stress time points, and the other for genes in the red, black and brown modules of the WGCNA analysis.

9.How to do the “Co-expression network analysis”? please state in the method.

Reply: Co-expression network analysis has been described in Materials and Methods.

**Reviewer #2:**

1. "The adaptation of plants to salinization is the joint result of a series of complex regulatory mechanisms, e.g., morphological, physiological, and genetic traits [1]." - Could you rephrase the sentence to clarify the role of these complex regulatory mechanisms in plant adaptation to salinity?

Reply: The expression of this sentence has been revised in the manuscript. The complex regulatory mechanisms of plant morphology, physiology, and genetic traits are the overall manifestation of adaptation to salinization

2."Under high-salt stress (high concentrations of salt), plant growth and development may be inhibited, and death is even possible." - What specific salt concentrations constitute "high-salt stress," and how does it affect plant growth and development?

Reply: The expression of this sentence has been revised in the manuscript. The expression "high" was removed from the manuscript.

3. "The presence of a large amount of Na+ and Cl- could cause ion toxicity and nutrient deficiency." - Can you explain how the presence of Na+ and Cl- ions leads to ion toxicity and nutrient deficiency in plants?

Reply: Salt ions can cause three types of stress on plants: osmotic stress, ion stress, and secondary damage^[1]^. Excessive accumulation of Na+ and Cl- ions in plant tissues causes serious ion imbalance in cells, affecting the absorption of nutrients such as K, N, P, Ca, Mg and Fe, and resulting in physiological and metabolic disorders, nutrient deficiency, growth retardation, and even death ^[2]^.

[1].Zhu JK. Abiotic stress signaling and responses in plants. Cell, 2016, 167:313-324.

[2]. Zhu J. Identification and mapping of qtl/genes for salinity tolerance in barley (*Hordeum vulgare* L.)[D]. Yangzhou university,2020.

4. "Most studies on the adaptation mechanisms of A. venetum under salt stress focus on physiological and phenotypic aspects, and some studies try to clarify the salt tolerance mechanism of A. venetum at the molecular level." - What have these studies revealed about the adaptation mechanisms of A. venetum to salt stress, both at the physiological and molecular levels?

Reply: The description of this sentence was revised in the manuscript to read: As for the adaptation mechanism of Apocynum apocynum under salt stress, some scholars have tried to clarify the salt tolerance mechanism of Apocynum apocynum at the molecular level. The adaptation mechanism of Apocynum apocynum to salt stress at molecular level was introduced in the introduction of the manuscript.

5. "Due to the accumulation of flavonoids, transgenic Arabidopsis thaliana showed higher salt tolerance than wild-type (WT) A. thaliana [11]." - Can you explain the relationship between flavonoid accumulation and salt tolerance in transgenic Arabidopsis thaliana?

Reply: To test this hypothesis, four flavonoid synthesis pathway genes, *AvF3H, AvF3′H, AvFLS*, and *AvF3GT*, were overexpressed in *A. thaliana*. The results found that the germination of transgenic plants were significantly better than the WT plants at 3 days under 100-mM NaCl stress. In addition, under 100-mM salt stress, the transgenic plants had longer roots than those of the WT plants. Based on these results, it is speculated that transgenic plants are more salt tolerant than wild-type plants, which may be related to the accumulation of total flavonoids.

6. • "The A. venetum DEAD-box helicase gene (AvDH1), stably inherited in the cotton genome, significantly improved the salt tolerance of transgenic cotton lines [12]." - What is the role of the AvDH1 gene in enhancing salt tolerance, and how does it interact with the cotton genome?

Reply: The yield of transgenic cotton is higher than that of control cotton, benefiting from the increase of boll number and boll weight^[3]^.

[3]. Chen J. Identification of salt tolerance in transgenic cotton with *Apocynum venetum* DEAD-Box Helicase Gene (*AvDH1*) [D]. Shandong Agricultural University, 2016.

4. • "This study analysed the transcriptome dataset of A. venetum under salt stress at six time points and identified core salt stress-responsive genes." - Can you provide more details about the methodology used in this analysis and the significance of identifying core salt stress-responsive genes in A. venetum?

Reply: To better understand the expression and functions of genes related to salt tolerance in transgenic cotton, we performed a digital gene expression (DGE) analysis between transgenic AvDH1 cotton and lu613. In nontransformed plants, 1359 DEGs were found following salt treatment. In comparison, 1150 DEGs were found in transgenic plants after salt treatment, 751 genes up-regulated in response to salt and 399 genes down-regulated. The results of qRT-PCR assays agreed with the DGE data, demonstrating the reliability of the transcriptome analysis.We found 35 transcription factors in the DEGs. Transcriptome profiling analysis showed that many key genes were involved in Phosphatidylinositol signaling system, peroxisome, ribosom e biogenesis in eukaryotes and RNA degradation,etc.

5. We identified 161 putative RNA helicase genes in the genome of the diploid cotton species Gossypium raimondii. We classified these helicases into three subfamilies, which include the DEAD-box, DEAH-box, and DExD/H-box gene families. The AvDH1 protein shows a sequence identity of 74% to GrDExD/H4 and GrDExD/H30. Syntenic analysis revealed that Gr DExD/H4 and Gr DExD/H30 had syntenic relationship; Transcriptome sequencing data demonstrated that Gr DExD/H4 and Gr DExD/H30 were expressed at high levels in all three samplesand may play important roles in various processes of Gossypium raimondii.

Reply: Digital gene expression (DGE) analysis was performed on transgenic AvDH1 cotton and acceptor lu613. The 161 putative RNA helicase genes had been identified in the genome of the diploid cotton species *Gossypium raimondii*. These helicases had been classified into three subfamilies, which include the *DEAD-box*, *DEAH-box*, and *DExD/H-box* gene families. The *AvDH1* protein shows a sequence identity of 74% to *GrDExD/H4* and *GrDExD/H30*. Syntenic analysis revealed that *Gr DExD/H4* and *Gr DExD/H30* had syntenic relationship. Transcriptome sequencing data demonstrated that *Gr DExD/H4* and *Gr DExD/H30* were expressed at high levels in all three samplesand may play important roles in various processes of *Gossypium raimondii*.

7. • "This study analysed the transcriptome dataset of A. venetum under salt stress at six time points and identified core salt stress-responsive genes." - Can you provide more details about the methodology used in this analysis and the significance of identifying core salt stress-responsive genes in A. venetum?

Reply: Quantitative proteomics of A. venetum using TMT technology under salt stress, the core protein obtained from differential protein network interactions at different stress times were mainly ribosomal proteins, indicating that the ribosomal proteins played an important role of A. venetum in response to salt stress.

8. • "The filtered clean reads ranged from 42975126 to 51285576, the total base number was 6150108311 to 7314153091, and the total base number was 6069861180 to 7301018739 after quality control." - There is a repetition of "the total base number." Please clarify this part.

Reply: The second“ the total base number” means the total base number after quality control. So the repetition of” the total base number” have different meanings. This issue in the manuscript has been revised.

9. • In the section about differentially expressed genes, you mentioned the p-value being less than 0.05 and a difference multiple greater than 2 as criteria. Could you explain the biological significance of these criteria in identifying differentially expressed genes?

Reply: The biological significance of the difference multiple greater than 2 means that the expression of the experimental group is more than twice up-regulated or down-regulated compared with the control group.

The P-value is a screening threshold to determine whether there are significant differences in gene expression between samples.

10.• Can you explain the biological implications of the observed gene expression patterns, such as the upregulation at early stages and downregulation at later stages of stress, in A. venetum under salt stress?

Reply: TRINITY_DN10560_c0_g1,photosystem II core complex proteins; TRINITY_DN6321_c0_g2,aquaporin PIP2-7; TRINITY_DN244_c0_g1,aquaporinPIP2-5; TRINITY_DN26308_c0_g1,ATPsynthase; TRINITY_DN10042_c0_g1,ABC transporter. These genes listed above all activated the response to stress in the early stage of *A. venetum*, with a tendency to up-regulate, and after late adaptation, the expression of these genes was down-regulated, indicating that these genes changed with the time of stress.

11. • In the GO classification and functional enrichment analysis, what are the functional categories that the differentially expressed genes are associated with, and how do these categories relate to A. venetum's response to salt stress?

Reply: Glucose metabolites can stabilize cell membranes and protoplasts, enhance osmotic potential against salt stress, and also play a role in supplying energy and carbon sources when photosynthesis is limited^[4]^.

Increasing the expression of antioxidant enzymes in plants under salt stress can reduce or avoid the damage caused by reactive oxygen species to a certain extent. Previous studies also found that the expression and activity of antioxidant enzymes in roots and stems of alfalfa seedlings were significantly increased under salt stress, which cleared active oxygen species in vivo and protected alfalfa seedlings from the harm caused by salt stress^[5]^.

The serine carboxypeptidase-like (SCPL) gene family plays an important role in the defense response of plants to abiotic stress and can regulate the defense mechanism of plants against adversity to resist the damage caused by adverse stress ^[6]^.

After plants are subjected to abiotic stress, carbon assimilation is inhibited, and the energy absorbed by plants cannot be fully utilized. Excessive energy accumulation causes reactive oxygen species (ROS) to accumulate in the reaction center of photosystem I(PSI) and photosystem II(PSII) in chloroplast thylakoids, causing photoinhibition and light damage to plants ^[7]^. Under stress conditions, photosystem II is very sensitive to reactive oxygen species and is easily damaged by reactive oxygen species ^[8]^. In addition, the correlation between functional classes of other core differentially expressed genes and salt tolerance of *A. venetum* was described in the discussion.

[4]. Li JunLing. Research on exploring the molecular mechanism of salt stress response in sugar beet based on multi-omics technology [D]. Harbin Institute of Technology, 2021

[5]. Xiong Junbo. Proteomic analysis of salt- responsive protein in *Medicago sativa* L. [D]. Chinese Academy of Agricultural Sciences, 2011.

[6]. Luo G. Study on the response mechanism of rice OsSCPD1 gene to drought stress [D]. Hunan Agricultural University, 2017.

[7]. Asada K.Production and Scavenging of Reactive Oxygen Species in Chloroplasts and Their Functions[J].Plant Physiology*,*2006,141(2):391-396

[8]. Murata N,Takahashi S,Nishiyama Y and Allakhverdiev SI.Photoinhibition of photosystem II under environmental stress[J].Biochimica et Biophysica Acta (BBA)-Bioenergetics, 2007,1767(6):414-421.

12. • Could you explain the biological significance of the identified pathways such as "zetin biosynthesis," "phenylpropanoid biosynthesis," and "flavonoid biosynthesis" in the context of A. venetum's response to salt stress?

Correction: "zetin biosynthesis" is correctly described as "Zeatin biosynthesis". The manuscript has been revised.

Zeatin is a natural cytokinin that occurs naturally in plants. The zeatin biosynthesis pathway in CrSNRK1a is upregulated according to the KEGG pathway, suggesting that zeatin may be involved in regulating salt tolerance in both varieties ^[9]^.

Flavonoids are closely related to the salt response and tolerance of plants, and salt stress can induce the expression of flavonoid biosynthesis genes and proteins ^[10]^.

The salt tolerance of Lonicera japonica was analyzed by TMT technology, and it was found that the genes related to phenylpropanoid biosynthesis and monoterpenoid biosynthesis pathway were up-regulated. Differential proteins were mainly involved in energy metabolism and carbohydrate metabolism pathway to enhance salt tolerance ^[11]^.

[9]. Yu bei.Effects of SNRK1a on the Physiological Mechanism of Rice under Salt Stress and Its Regulating Measures [D]. Jilin Agricultural University,2023

[10]. Jiang JY, Zhu S, Zhang Y, et al. Integration of them lipidomic and transcriptomic profiles  reveals will genes and regulatory mechanisms of Schizochytrium sp. in response to salt stress. [J]. Bioresource Technology, 2019,249.

[11]. Cai Z C. Study on the quality formation of lonicerae japonicae flos under salt stress based on multi omics [D]. Nanjing University of Chinese Medicine,2021.

13. •When you mention "reverse transporter activity (GO:0015297)," could you clarify what this term means in the context of salt stress and its relationship to salt adaptation?

Correction: “reverse transporter activity” is correctly described as “antiporter activity”. The manuscript has been revised.

Na+/H+ antiporter is the most important transporter for plants to adapt to high salt environment. Na+/H+ antiporter located on the plasma membrane can expel Na+ from the cytoplasm and make it enter the symplast or culture medium. Na+/H+ antiporter located on the vacuole membrane can segregate Na+ from the cytoplasm into the vacuole ^[12]^.

[12]. Hunte C, Screpanti E, Venturi M, Rimon A, Padan E, Michel H. Structure of a Na^+^/H^+^ antiporter and insights into mechanism of action and regulation by pH [J]. Nature, 2005, 435: 1197-1202.

14. •What is the significance of the turquoise module containing the most genes and the green-yellow module with the smallest number of genes in the co-expression network analysis?

Reply: WGCNA analysis first calculates the correlation coefficient between the genes and then classifies the genes according to their expression patterns. The genes with similar expression patterns are grouped into a module, that is, genes with similar functions are divided into the same module. As a result, different modules contain different numbers of genes. Therefore, In this study, the turquoise module contained the most genes, with 6274 genes. The module with the smallest number of genes is the green-yellow module, with 110 genes.

15. • How do the identified modules and their correlations with differentially expressed genes relate to the plant's response to salt stress, and what specific functions or processes are associated with each module?

Reply: We conducted GO enrichment analysis for each module gene, and found that the gene functions in red, brown and black were closely related to salt stress, so we mainly focused on the genes in these three modules.

16. • Can you elaborate on the relationship between the correlation coefficients and the identified modules in the context of A. venetum's adaptation to salt stress?

Reply: The correlation coefficient is used to indicate the strength of the relationship between the module and the phenotype. The larger the correlation coefficient, the smaller the P-value of significance, indicating the stronger the correlation between the module and the phenotype.

17. • In the GO enrichment analysis, how do pathways related to salt stress, such as "water transmembrane transporter activity," "reverse transporter activity," and "response to reactive oxygen species," contribute to A. venetum's salt stress response, and what are the key genes associated with these pathways?

Correction: Change “water transmembrane transporter activity”to “water channel activity (GO:0015250)” The genes included in the water channel activity pathway are TRINITY_DN6321_c0_g2 and TRINITY_DN244_c0_g1.

Aquaporins are involved in osmoregulation in plant cells ^[13]^, improve the hydraulic conductivity of roots and leaves ^[14]^, enhance transpiration ^[15]^, and effectively improve plant water use efficiency under abiotic stress conditions. Transgenic bananas overexpressing *MusaPIP2;6* exhibited better photosynthetic efficiency and lower membrane damage under salt stress ^[16]^.

Abiotic stress leads to excessive production of reactive oxygen species (ROS) in plants, which on the one hand causes damage to cell components and on the other hand can act as a signal molecule of stress response ^[17]^.Plants can regulate ROS levels through complex mechanisms, such as clearing ROS with ascorbate peroxidase (APX), glutathione peroxidase (GPX), glutathione S-transferase (GST), and peroxide dismutase (POD).In this study, peroxidase (POD) and ascorbate peroxidase both showed the same trend, that is, the protein quantity and expression increased with the extension of stress time. The result was consistent with the change trend of POD content in the previous physiological index determination.The upregulated expression of POD and APX in mangrove under salt stress is consistent with the significant increase of POD, SOD, APX and CAT protein abundance in mangrove (*Kandelia candel*) under high salt stress ^[18]^. Therefore, the upregulation of these proteins involved in REDOX homeostasis implies that *A. venetum* activates ROS clearance mechanisms to reduce salt-induced oxidative stress and thus enhance salt tolerance.

The genes involved in “response to reactive oxygen species” pathway include TRINITY_DN17703_c1_g1、TRINITY_DN11463_c0_g1、TRINITY_DN16417_c0_g1、TRINITY_DN4886_c0_g1、TRINITY_DN7443_c0_g2、TRINITY_DN85189_c0_g1 and TRINITY_DN10833_c0_g1.

The function of the antiporter activity has been answered in question 13, and it will not be repeated here. The genes included in this pathway include TRINITY_DN2615_c0_g1、TRINITY_DN8915_c0_g1、TRINITY_DN29574_c0_g1、TRINITY_DN5877_c0_g1、TRINITY_DN9429_c0_g4 and TRINITY_DN1648_c0_g1.

[13]. Wallace IS, Choi WG, Roberts DM. The structure, function and regulation of the nodulin 26-like intrinsic protein family of plant aquaglyceroporins[J]. Biochimica et Biophysica Acta (BBA) -Biomembranes, 2006, 1758(8): 1165-1175.

[14]. Siefritz F, TyreeMT, Lovisolo C, et al. PIP1 plasma membrane aquaporins in tobacco from cellular effects to function in plants[J]. Plant Cell, 2002, 14(4): 869-876.

[15]. Sade N, Gebretsadik M, Seligmann R, et al. The role of tobacco Aquaporin1 in improving water use efficiency, hydraulic conductivity, and yield production under salt stress[J]. Plant Physiology, 2010, 152(1): 245-254.

[16]. Sreedharan S, Shekhawat UK, Ganapathi TR. Constitutive and stress-inducible overexpression of a native aquaporin gene (*MusaPIP2; 6*) in transgenic banana plants signals its pivotal role in salt tolerance[J]. Plant Mol Biol, 2015, 88(1-2): 41-52.

[17]. Sun H. Analysis of the molecular mechanisms of cotton response to salt stress[D]. Central China Agricultural University, 2019.

[18]. Wang X, Gu Y, Zhang X, et al. Bioinformatics and stress response expression analysis of poplar HSF family genes[J]. Journal of Beijing Forestry University, 2021, 43(2): 34-45.

**Reviewer #3:**
Need some minor suggested improvements
1. In the Material and Methods section, kindly ensure to explicitly mention the tool or software utilized for the calculation of FPKM in the determination of gene expression values

Reply: FPKM and read counts value of each unigene was calculated using bowtie2 (Version 2.3.3.1) and eXpress (Version 1.5.1).

2. The Material and Methods section appears to be incomplete. We kindly request you to incorporate the following:
• Clearly state the developmental stage of the plant at which the sample was collected for transcriptome analysis

Reply: The experimental material used in this study were Annual A. venetum seedlings.

• Provide a detailed introduction to Co-expression network analysis, along with pertinent information regarding related packages such as WGCNA, in the Material and Methods section.

Reply: We have added a detailed description of the WGCNA analysis to the manuscript in accordance with the reviewer's comments.

• Elaborate on the techniques and tools employed for the formation of Gene Ontology annotations

Reply: GO enrichment and KEGG pathway enrichment analysis of DEGs were respectively performed using R based on the hypergeometric distribution.

3. For enhanced clarity and comprehension, we recommend including a supplementary table presenting the correlation coefficients of gene pairs derived from the WGCNA analysis.

Reply: We have added the supplementary table in the appendix 1.

4. Please provide a comprehensive explanation and specification of the twelve modules obtained from the WGCNA analysis.

Reply: We screened the genes of 12 modules and selected the three modules of focus: red, black and brown. We have added the information of 12 modules in the appendix 2.

**Reviewer #4:**
The authors have made an interesting research entitled “Identification of core genes involved in the response of Apocynum venetum to salt stress based on transcriptome sequencing and WGCNA”.
They studied some molecular mechanisms of salt tolerance in A. venetum and introduced some key genes and gene networks that are involved in salt stress regulation and salt tolerance mechanisms in plants.
One concern is about gene IDs. Replace the gene IDs like TRINITY_DN102_c0_g1, TRINITY_DN3073_c0_g1 and TRINITY_DN6732_c0_g1 with GenBank accession numbers or convert those to Arabidopsis orthologues
I think this article is worthy to be published.
Regards,

Reply: We converted the gene ID that we focused on in the manuscript to the Arabidopsis orthologues gene, and finally only succeeded in comparing two genes. Therefore, gene ids continue to be used for representation in the manuscript.

TRINITY_DN7962_c0_g1_i1_3 (AT3G19820), TRINITY_DN2135_c0_g1_i2_7 (AT4G30130).
